# Smartphone Apps for Containing the COVID-19 Pandemic in Germany: Qualitative Interview Study With Experts Based on Grounded Theory

**DOI:** 10.2196/45549

**Published:** 2023-10-20

**Authors:** Dennis Krämer, Elisabeth Brachem, Lydia Schneider-Reuter, Isabella D'Angelo, Jochen Vollmann, Joschka Haltaufderheide

**Affiliations:** 1 Faculty of Social Sciences Georg-August-University Göttingen Göttingen Germany; 2 Faculty of Medicine Ruhr-University Bochum Bochum Germany; 3 Faculty of Health University of Witten/Herdecke Witten Germany; 4 Joint Faculty of Health Sciences Brandenburg University of Potsdam Potsdam Germany

**Keywords:** Corona-Warn-App, COVID-19 pandemic, eHealth, Germany, health technology, mobile phone, qualitative research, sovereignty, transparency

## Abstract

**Background:**

Smartphone apps, including those for digital contact tracing (DCT), played a crucial role in containing infections during the COVID-19 pandemic. Their primary function is to generate and disseminate information to disrupt transmissions based on various events, such as encounters, vaccinations, locations, or infections. Although the functionality of these apps has been extensively studied, there is still a lack of qualitative research addressing critical issues.

**Objective:**

We will demonstrate that the use of DCT presents a challenge due to the tension between continuous health monitoring and uncertainties related to transparency and user sovereignty. On one hand, DCT enables the monitoring of various risk factors, including data-based calculations of infection probabilities. On the other hand, continuous risk management is intertwined with several uncertainties, including the unclear storage of personal data, who has access to it, and how it will be used in the future.

**Methods:**

We focus on the German “Corona-Warn-App” and support our argument with empirical data from 19 expert interviews conducted between 2020 and 2021. The interviews were conducted using a semistructured questionnaire and analyzed according to the principles of grounded theory.

**Results:**

Our data underscores 3 dimensions: transparency, data sovereignty, and the east-west divide. While transparency is considered an essential foundation for establishing trust in the use of DCT by providing a sense of security, data sovereignty is seen as a high value during the pandemic, protecting users from an undesired loss of control. The aspect of the east-west divide highlights the idea of incorporating sociocultural values and standards into technology, emphasizing that algorithms and data-driven elements, such as distance indicators, encounters, and isolations, are also influenced by sociocultural factors.

**Conclusions:**

The effective use of DCT for pandemic containment relies on achieving a balance between individual control and technological prevention. Maximizing the technological benefits of these tools is crucial. However, users must also be mindful of the information they share and maintain control over their shared data.

## Introduction

### Background

During the COVID-19 pandemic, governments worldwide expanded their use of digital technologies as part of their pandemic health policies [1,2]. Among the most widespread were those that facilitated population-wide health monitoring through smartphone programs, such as apps for digital contact tracing (DCT). Most governments developed DCT apps in the early stages of the pandemic to combat SARS-CoV-2 by continuously aggregating and disseminating personalized information about the users, including their locations, encounters, infections, and vaccinations [3]. Unlike certain Asian countries [4], the majority of DCT apps developed in the West adhere to a voluntary approach. Individuals can choose to carry their smartphones, download an app, and share information about infections or vaccinations with others. Simultaneously, the possibility of voluntary participation and self-determined consent brings with it the challenge that users have certain expectations regarding how their data are stored, who has access to it, and how it will be used in the future [5]. In this regard, several studies have already shown that factors such as sovereignty and transparency play crucial roles in the acceptance of DCT apps [6].

However, there is a lack of qualitative research that can provide more precise insights into how various uncertainties connected to the use of DCT apps are evaluated and perceived. Our empirical data make it clear that, under the condition of a voluntary approach, the challenge is rooted in a tension between the potential of continuous smartphone-based monitoring and the various uncertainties associated with the use of DCT. On one hand, DCT enables the collection of a variety of risk factors, including the data-based calculation of infection probabilities. On the other hand, digital risk management is associated with several sensitive issues, such as a lack of understanding regarding where personal data are stored and how it will be used in the future.

To provide a detailed description, we will begin by outlining the functionalities of DCT using the example of the German “Corona-Warn-App” [CWA]. We then share the results of a qualitative study based on 19 expert interviews conducted between 2020 and 2021. Based on this, we will explain how the 3 dimensions—transparency, data sovereignty, and the east-west divide—are crucial for the use of DCT.

### Framework of CWA

**Figure 1:**
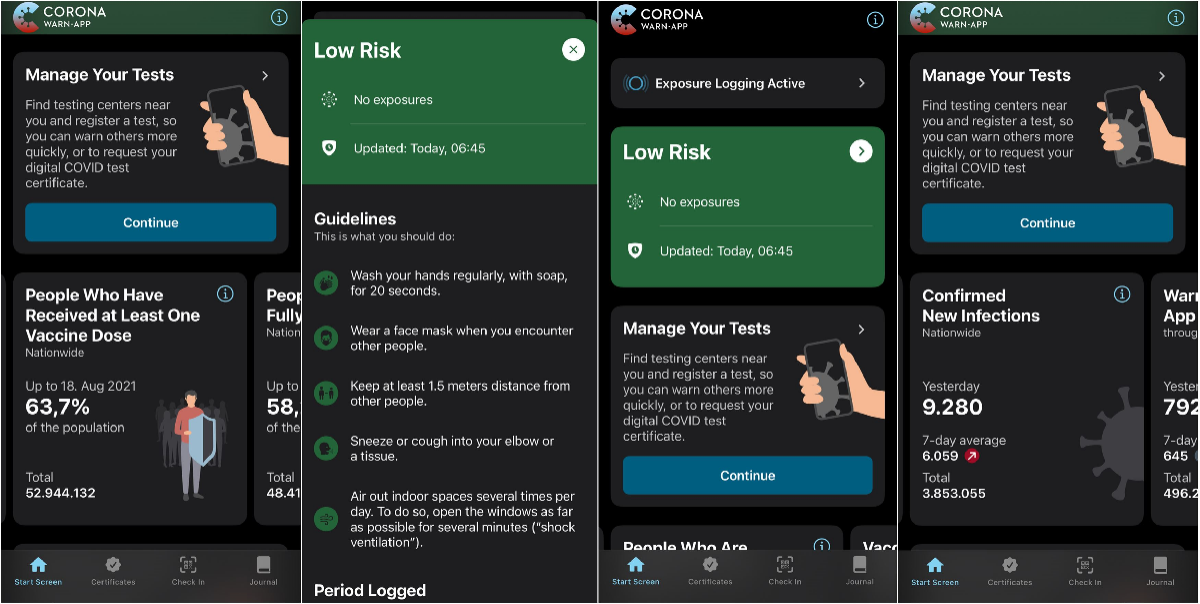
Overview of the Corona-Warn-App.

The CWA is the outcome of a collaborative effort between the German government and various German research institutions and companies, including Systeme, Anwendungen und Produkte in der Datenverarbeitung and Deutsche Telekom. Officially launched by the Robert Koch Institute on June 16, 2020, it was designated as a “building block of pandemic control” [7]. In addition to contact restrictions, requirements for wearing face masks, and limitations on visitor numbers in places such as restaurants and supermarkets, the German government considered the CWA a crucial element of its digital strategy to combat the pandemic. The functionalities of the CWA include sharing positive test results, uploading vaccination certificates, calculating risk groups, personalized check-ins based on QR codes, maintaining a contact diary, and accessing real-time information, such as hospitalizations, incidence, and vaccination rates. Figure 1 provides a general overview of the various functionalities of the CWA, including the risk communication.

In accordance with Germany’s stringent data laws, the CWA operates on a voluntary basis [8]. This means that users must agree to download the app, activate the Bluetooth function on their mobile devices, or share a positive test result. Furthermore, data are stored in a decentralized and anonymized manner on users’ respective smartphones without being transmitted to an external server.

### Framework of DCT

The primary function of the CWA is contact tracing, which is a well-established containment strategy in the history of pandemics. Scholars, such as the French philosopher Michel Foucault [9], have examined contact tracing in the Western world by drawing parallels to historical examples such as leprosy, smallpox, and the plague. Foucault [9] interpreted these pandemics as specific indicators for understanding contemporary power relations, including those between citizens and rulers or governments. According to this perspective, during the Middle Ages, individuals with leprosy were often excluded from the community and banished to the outskirts of cities and villages. Their exclusion was closely monitored, and they were prohibited from reintegrating into society. In the efforts to combat the plague, people were assigned specific areas within the cities and villages. These locations were then divided into zones based on the level of risk. During the smallpox epidemic, for the first time, the entire human population was treated as a statistical collective. Infections, symptoms, and disease progression were considered alongside other factors such as gender, nationality, and age to develop comprehensive containment strategies: “the leper gave rise to rituals of exclusion… the plague gave rise to disciplinary diagrams” [9]. In this historical analysis, DCT represents an exemplary approach to managing pandemics in contemporary times. Individuals are no longer simply classified as threats and excluded from specific areas. Instead, they proactively warn and monitor themselves, transforming their numerous interactions into trackable information.

Technically, encounters are simulated through the exchange of Bluetooth signals, which are then recorded as random codes on smartphones for 14 days (assuming the incubation period) [10]. If users receive a positive test result, they have the option to transmit it anonymously to a server. Other smartphones regularly download the information stored on this server and cross-reference it with their own randomly generated codes to identify previous encounters with individuals who have tested positive for COVID-19. When a match is found, a transmission risk is calculated. This calculation involves 3 variables: distance, duration of the encounter, and the number of days since the positive test occurred. The CWA subsequently calculates a risk score, which is used to determine a risk notification.

- A “risk notification” appears when a contact lasting ≥10 minutes takes place at an average distance of ≤3 meters, when the contact shares a positive test result, and when the test result is not older than 14 days.
- The “risk determination” results from an ongoing quantification that combines the 3 aforementioned variables and categorizes users on a 3-level scale: unknown risk, low risk, or increased risk.

## Methods

### Overview

Under the condition of voluntary participation, several questions arise. How can users of DCT ensure that their sensitive data will not end up in the wrong hands and be further processed by third parties, such as companies or health insurance providers? How can they be certain that sensitive information, such as a COVID-19 infection, is truly being captured anonymously, will be promptly deleted, and will not result in long-term consequences?

### Research Project “ELISA”

The project “The Ethics of Live-Tracking Applications in Connection with SARS-CoV-2” (ELISA; grant 01KI20527) aimed to address sensitive issues related to the use of DCT through a qualitative expert survey. It received funding from the German Federal Ministry of Education and Research (BMBF) and took place from October 2020 to December 2021. Its primary focus was on gaining a deeper understanding of social and technical aspects, including the algorithmic calculation of infection probabilities, the assessment and quantification of users as risk factors, and the storage and processing of shared data. To achieve its objectives, the project was divided into 2 main subprojects: an empirical subproject and an ethical subproject. The empirical subproject aimed to create a qualitative database to highlight critical points associated with the implementation of DCT. The ethical subproject focused on capturing the qualitative research findings and subjecting them to ethical analysis [11,12].

### Study Design

#### Overview

**Figure 2:**
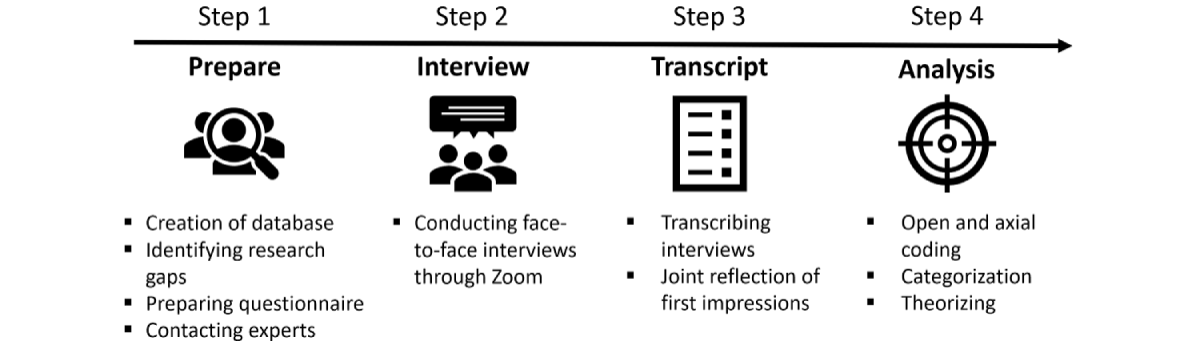
Steps of the empirical analysis.

Both subprojects worked closely together, enabling the completion of various tasks by a 5-member team. These tasks included a literature review, questionnaire design, data analysis, and subsequent work on publications. The team consisted of 2 social scientists and 3 ethicists. A particular challenge was that, at the beginning of the project, there was limited research on DCT in the context of the COVID-19 pandemic. Furthermore, the project’s duration of 12 months was relatively short for pioneering work aimed at empirically and ethically analyzing the significance of DCT for pandemic control. Given this context, there was a challenge in initiating a process to identify relevant research aspects in unfamiliar territory. To achieve this, a 4-step research process was used. Figure 2 provides an overview of the 4 steps of the empirical analysis.

#### Step 1

Initially, we created a database to gain an overview of previous publications on DCT. Our goal was to identify research gaps and sensitive topics. These publications were sourced from various databases, including Web of Science, ProQuest, Scopus, Google Scholar, and arXiv. Since the project commenced shortly after the outbreak of SARS-CoV-2, some research findings were in the form of preprints. We organized these publications using reference management software (Citavi; Swiss Academic Software) and distributed them among team members. Each team member then generated summaries to create an overview. The research questions and identified gaps were documented and subsequently discussed by the team. Based on this, we developed an initial categorization system, which we iteratively refined as new publications emerged. This categorization system consists of 4 meta-categories, each containing 3-4 subcategories. To further explore the research gaps and the intended meta- and subcategories, we designed a semistructured questionnaire. The questionnaire focused on the meta- and subcategories, featuring several questions (2-3 per subcategory). Following this, we conducted a validation testing phase to identify weaknesses and missing connections within the questionnaire. For this purpose, we presented the questionnaire during a web-based workshop attended by approximately 15 researchers from various disciplines, including social science, computer science, medicine, and ethics. Additionally, representatives from the Robert Koch Institute were present. We initiated contact with these researchers through email to secure their participation in empirical and theoretical DCT research. The discussions during this workshop were documented and informed the final design of the questionnaire. Table 1 provides an overview of the empirical research areas and lists selected questions.

**Table 1:** The ethics of live-tracking applications in connection with SARS-CoV-2 questionnaire.

Areas	Focus areas	Questions (examples)
Transparency	Self-determination Data control Information Asymmetries	What do you think about how transparent the process of collecting, sharing, and archiving user data is? How self-determined do you consider the way users can manage their data?
Privacy	Uncertainties Commitment Personal data Sensitive information	What is your opinion on the mandatory use of the DCT^a^ app? How do you evaluate the way sensitive information, such as risk scores, infections, or positive COVID-19 results, is communicated to users?
Justice	Barriers Discrimination Collective protection vs individual freedom	Do you see any barriers regarding access to and the use of the app? Are certain groups particularly at risk of exclusion or disadvantage?
Epidemiology	Efficiency Risk calculation Data relevance	What do you think about the classification of hierarchical risk groups? Would it make sense to collect additional data for effectiveness or to combine it with other user data?

^a^DCT: digital contact tracing.

#### Step 2

Between November 2020 and April 2021, a total of 19 face-to-face interviews were conducted with scholars from various disciplines (details in the “Research Participants” section). Due to social distancing measures, all interviews were conducted through Zoom. One criterion for selecting interviewees was that they were conducting research related to the CWA, regardless of whether their research focused on Germany or other countries. The interviews were exclusively conducted by 1 team member who was responsible for the empirical subproject. A total of 16 interviews were conducted in German, and 3 were conducted in English. For the English interviews, the questionnaire was translated by a native English speaker. This also applies to the interview excerpts quoted in this paper. The interview duration varied considerably, ranging from 21 to 81 minutes. Reasons for this variation can be attributed to the individual response behavior of the respondents. Regardless of their academic backgrounds, the average length of interviews with researchers from critical-reflective disciplines, such as social sciences and ethics, was 52 minutes. This is comparable to researchers from technical and natural science disciplines, such as medicine and computer science, where the average length was 50 minutes. Both the shortest interview, lasting 21 minutes, and the longest interview, lasting 81 minutes, were conducted with postdoctoral researchers in the field of computer science. During the interviews, we maintained a balance between providing open-ended questions and using structured interview guidance. We ensured that the interviews followed the semistructured interview guide, which allowed for follow-up questions and additional exploration. Additionally, at the end of the interviews, respondents had the opportunity to address any points they felt were missing. This flexibility allowed the questionnaire to evolve throughout the research process.

#### Step 3

The interviews were recorded on Zoom and transcribed, and the transcriptions were subsequently discussed in regular data sessions with project members. The goal of these data sessions was to collaboratively analyze the transcripts, addressing the research gaps identified at the beginning of the project. Additionally, these sessions aimed to uncover and explore previously unknown aspects. This ongoing process contributed to the continuous development of the questionnaire and the inclusion of additional questions during the data collection and joint reflection phases. Concurrently, preliminary findings were presented and discussed during a web-based meeting organized by the BMBF in January 2021, as well as within the framework of an interdisciplinary workshop organized by our team in September 2021. The events primarily focused on topics such as the technical implementation of solidarity and sensitive issues related to user participation, awareness, and informed consent [13]. Workshop participants included members of the project team as well as researchers from various fields who were involved in projects related to the CWA.

#### Step 4

##### Analysis Process

The data analysis is based on the principles of inductive theory generation, following the grounded theory [14]. Starting from the perspective that explanatory approaches are grounded in empirical data and need to be elaborated, we used a systematic, iterative approach consisting of 4 steps.

##### Data Collection

Initially, systematic data collection was conducted based on the specified criteria, with a focus on the phenomenon under study. The aim was to comprehensively understand it without any preconceived assumptions. The main objective of this step was to continuously collect new data on the phenomenon and integrate the findings into subsequent surveys to further investigate focal points in more depth.

##### Coding

The second step involved reading the transcribed interviews without any preconceptions and dividing the identified challenges, focal points, and issues into smaller segments (open coding). Subsequently, these segments were linked together to identify patterns and establish deeper connections (axial coding). Regular team meetings, data sessions, and workshops were used for discussions, integration, deletion, renaming, or elaboration of the codes.

##### Categorization

**Figure 3:**
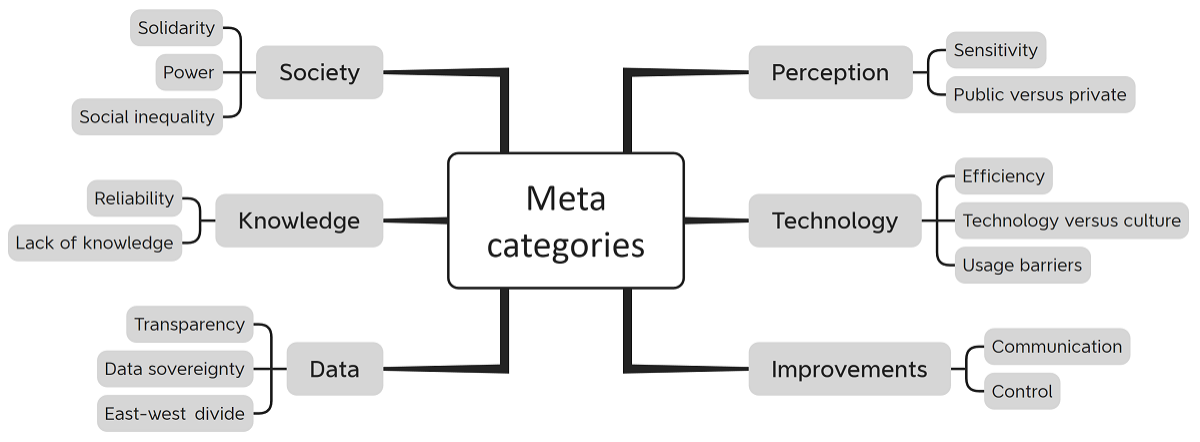
Overview of the meta- and subcategories.

The coding was then transformed into 6 meta categories and 15 subcategories, each labeled with concise names to identify their content-related connections. The reason for the categorization differing from the categories in the interview guide is that, as is typical of qualitative interviews, respondents may also address additional and entirely different points when answering the questions. Figure 3 provides an overview of the meta- and subcategories.

##### Theorizing

Through this iterative coding process, the categories were progressively refined by incorporating additional data. This allowed for the development of more comprehensive explanatory approaches to specific topics. The individual categories were subsequently described in writing and ultimately incorporated into the broader scientific discourse on DCT.

### Research Participants

In order to maximize learning within the limitations of the project’s short duration and the worldwide implementation of DCT apps for pandemic control, an expert survey was conducted. Our goal was to identify competent experts who could provide scientifically sound explanations for technical, legal, societal, and medical challenges related to the CWA. Experts were defined as scholars from various scientific fields who had already published or given talks about DCT. At the outset of the project, we conducted internal research to identify relevant experts, ensuring that we formed an interdisciplinary group. Additionally, we presented the project at conferences and institutions, including medical associations. To achieve this, individuals were contacted through email and provided with a brief project description. Out of a total of 33 individuals contacted, 19 participated in the study, and among them, 17 were affiliated with German universities, 1 with a Dutch institution, and 1 with a British institution. Overall, 8 of the participants were female, and 11 were male. Table 2 provides an overview of the research participants and their professional backgrounds.

**Table 2:** Sample expert survey.

Number	Gender	Research field
1	Male	Journalism
2	Male	Ethics
3	Male	Ethics
4	Male	Computer science
5	Male	Ethics
6	Male	Legal studies
7	Female	Social science
8	Female	Medicine
9	Female	Social science
10	Female	Medicine
11	Male	Legal studies
12	Male	Computer science
13	Female	Medicine
14	Female	Ethics
15	Female	Medicine
16	Male	Computer science
17	Male	Computer science
18	Male	Social science
19	Female	Ethics

### Ethical Considerations

The empirical part of this study was conducted in accordance with the principles of the Declaration of Helsinki. Due to the sensitivity of pandemic research, approval was obtained from the ethics committee of Ruhr-University Bochum, Germany (14.10.2020/20-7061-BR). Informed consent was obtained from all participants included in the study. Informed consent covered sensitive and private information. All participants voluntarily engaged in the research activities and had the option to withdraw at any time. The participants did not receive any financial compensation for their participation in the study. Interviews and transcribed qualitative data are stored on an external storage medium at Ruhr-University Bochum and can be made available upon request.

### Results Interpretation

#### Transparency

**Figure 4:**
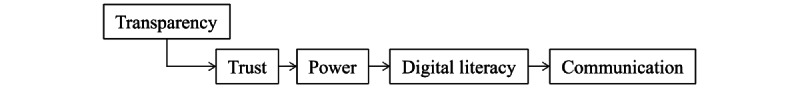
Overview of different dimensions of transparency.

Transparency is a widely debated topic in media studies. Renowned scholars, such as Jean Baudrillard [15], describe transparency in digital communication as an essential element of an “ecstatic flow of information” and a necessity for individuals to perceive themselves as sovereign in an increasingly complex media landscape. The importance of transparency has also been emphasized in the context of DCT. It has been emphasized that transparency is necessary when people need to be convinced of the app’s usefulness, particularly when its use is voluntary [6]. For instance, Milan [16] has clarified that the low acceptance of the US DCT app by the general public is mainly due to a “lack of transparency.” This corresponds to the position described by Torilli and Floridi [17], where they assert that transparency is a prerequisite for conveying to users the sense of rational decision-making. Our data reveal additional insights into the importance of transparency. Figure 4 indicates the different dimensions of transparency resulting from the qualitative data.

In essence, transparency is considered a “protective function” (interview [IV]-4) and a “basis of trust” (IV-2) that plays a crucial role in enabling public participation in the digital pandemic response. In the context of DCT deployment, transparency is defined as providing information that promotes trust in the use of technologies. It ensures that civil society can scrutinize the use of these technologies and guarantees that users give their consent for the release of sensitive information, such as location and health data. In this context, our interviews reveal an additional dimension that can be described as an information-based power relationship. It involves not only being informed about which data are stored in what situation and for how long, but also being informed about who has access to these data and how they might potentially be processed in the future. In this regard, transparency entails not only disclosing the processes of data collection and dissemination but also understanding the various interest groups and their intentions. Thus, transparency serves as a means to assert sovereignty and control over one’s data.

Who has what data now, what do they need now, and why do they need it? I believe that this creates a great deal of uncertainty and that you still want to protect your data and that things are accessed way too often, where you say: ‘Why am I still getting advertising for it now, just because I searched for it once?’

IV-7

A good example I find at this point is that people say, ‘Well, now we have the app, and what will it be able to do in the future?’ It is a bit like the vaccination card. Now we register for the Corona vaccination, and what do I have to register for in the future? Do I then enter HIV? Do I enter hepatitis? […] And these are things that I find justifiable for people to criticize.

IV-9

Furthermore, it is emphasized that transparency always presupposes a certain level of “digital literacy” (IV-7) and “previous knowledge” (IV-14) to understand the disclosed processes. In this context, transparency can pose a challenge for individuals. On one hand, it aims to comprehensively explain the mechanism of digital pandemic control. On the other hand, applications like DCT apps are highly complex programs that require expertise to understand processes such as risk calculation using algorithms.

Concerning the app, I think the representation of transparency is really a complicated story. This may always be difficult with apps because you need so much prior knowledge […] to understand the whole thing. It simply goes far beyond what you can understand as a normal consumer.

IV-14

Finally, transparency requires clear communication about transparency. The interviewed experts note that, while the CWA does indeed adhere to stringent data protection regulations in Germany, it also publicly communicates the aforementioned calculation and storage processes on their websites. At the same time, however, users are often not informed about where they can find this information or how to understand it. In order to obtain consent based on transparency, it is essential for transparency to not only exist but also be effectively communicated in a focused manner.

So, basically, transparency is available, but, on the other hand, there is not enough transparency so that the transparency is present. […] Here is a link where you can click to see how the app is used, how everything is presented transparently, and where the app’s problems are. Many people do not know how or where to get information about this.

IV-15

#### Data Sovereignty

The significance of sovereignty in digital communication has received much attention in current scientific debates [18,19]. Various developments in recent years, such as the growing influence of social networks, the proliferation of internet-of-things technologies, and the continuous advancement of artificial intelligence, raise the question of how individuals can act as sovereign users of devices and programs in the face of the increasing influence of technology in all aspects of life [20,21]. At the same time, there are only a few works that address conceptual issues and inquire about what sovereignty in digital communication specifically entails. Floridi [22] describes “digital sovereignty” as a complex network consisting of different actors, each with their interests. Similar approaches have recently been examined in the context of the international deployment of DCT apps [23]. Following Floridi [22], Tretter [24] illustrates that this network is determined by 3 actors: the governments, each of which develops its own DCT app while establishing individual standards for data storage and digital surveillance; the tech companies, such as Google and Apple, which provide, but can also withdraw, the digital infrastructure to implement these apps and thus essentially have access to the data generated through smartphones; and the individuals who are at the core of DCT and whose behavior forms the basis for digital pandemic response.

This study supports another point that we refer to as data sovereignty. Against this background, sovereignty extends the abovementioned aspect of transparency to include the importance of control over one’s data on the part of DCT app users. Data sovereignty, therefore, goes beyond the realm of privacy (eg, data protection). It extends to the aspects of insight and understanding, empowering individuals, and aiming to counter data exploitation, including criminal access (hacking, identity theft, and data breaches) and economic exploitation. During a pandemic, data sovereignty takes on special importance as individuals not only share sensitive information but also experience a sense of powerlessness in the face of an external threat.

But, of course, that also means that this data exists and ultimately no system is unhackable.

IV-10

One suddenly becomes an ally in the fight against a pandemic and, thus, also cedes a considerable part of sovereignty, necessarily.

IV-11

In this context, it became evident that the interviewed experts were less focused on theoretical reflection and more concerned with the practical implementation of sovereignty. They were particularly interested in addressing the question of how data sovereignty can be achieved. In this context, primarily technical measures were mentioned, such as data encryption, dissemination, and storage. It was emphasized in nearly all interviews that the CWA operates as a model example of third-party access, as it stores the generated data on smartphones and forwards encrypted encounters with others to a server.

Firstly, the data remains in the operating system, and it is also encrypted. Therefore, every mobile phone changes its ID every 15 minutes, so you also need various encryption keys to track even a single mobile phone. This means that if my phone hits your phone more than once, it will not show up with the same ID in my phone.

IV-10

I think the main advantage is that you can trust this decentralized approach blindly because nothing leaves your mobile phone, eg, no data and information, until you decide at some point and say, I would now like to inform my contacts that I have been tested positive.

IV-16

#### East-West Divide

In the code strand “east-west divide,” experts emphasize that DCT apps not only serve as efficient tools for pandemic control but also incorporate sociocultural values and standards. These positions draw on critical research approaches that describe the deployment of DCT in Asian countries, such as China, South Korea, and Taiwan, as “unnecessarily harmful to human rights” [25] and accuse them of a “lack of a reliable regulatory backbone on public data collection” [26]. Against this backdrop, governments in these countries tend to use DCT as a collectivist technology, legitimizing aspects such as quarantine monitoring or personalized data collection with the aim of protecting society. In Western media, this approach is often described not only as collectivist but also as autocratic [27]. It is well-documented that in certain East Asian states, DCT serves as just one component of a comprehensive surveillance network. This network includes various digital elements, such as automated teller machines, surveillance cameras, drones, and digital patient records, which communicate with each other. This seamless communication enables the recording of interactions and the tracking of individuals’ locations.

Given this background, experts consider DCT not only a matter of technical design and functionality but also a subject of political, legal, and medical discourses that influence the development of such technology. On one hand, the digital pandemic control measures implemented in countries like China or Taiwan are often considered highly invasive and are often contrasted with what is known as the “Western approach.”

So perhaps the best example is China. A low number of cases. However, the state also knows where every citizen goes, of course. They have detailed profiles. The state can use a report to entire quarantine districts or restrict movement. However, this is also not in line with European values.

IV-8

On the other hand, the “Asian approach” is regarded as a model for an effective pandemic response.

You can look at China, Japan, Korea, etc. Then you can say these tracking systems definitely work, but they are also quite controversial. Moreover, now I am looking at China. Especially in autocratic, rather dictatorial states, in states that already rely heavily on surveillance and monitoring systems, this technology is not entirely new for citizens.

IV-9

However, the Western response to the pandemic also compels us to critically question this perspective. In the early stages of the pandemic in Adelaide, Australia, 2 individuals from China who were infected with SARS-CoV-2 were quarantined. In addition, their movements were monitored through their smartphones, and the location data were transmitted to the local police. Similar cases also occurred in Germany. To keep stores and restaurants open, owners were mandated to record the contact information of all visitors. The responsibility for data collection rested with the respective establishments, with the understanding that the data would be collected solely for informational purposes, such as contact tracing in the event of contact with a person who tested positive for COVID-19. Contrary to this assurance, in some cases, these data were used for other purposes, such as police investigations [28].

The typical example in the restaurants, where people enter their names in the lists, is that many people have entered false names or false contact details because there have been cases in Bavaria, for example, where the police have used these lists for investigative procedures, and I think that where the data protection concerns come from.

IV-17

As a result, some citizens objected to disclosing their data and considered adopting strategies to evade it. One of these strategies was to falsify the data in order to avoid government interference.

An interesting thought is when you start opening restaurants again. Then, at least over the summer and autumn, they still worked with a very analog system, meaning that people entered papers, wrong telephone numbers, wrong addresses, etc

IV-12

## Discussion

### Conclusions

The COVID-19 pandemic represents one of the most significant global challenges of the early 21st century. Paradigmatic of the digital age, governments in nearly all countries have developed technologies for digital pandemic control, with DCT apps playing a central role in implementing comprehensive health surveillance. This qualitative study has demonstrated that several aspects are of central importance under the condition of voluntary use of these apps. These include transparency regarding the processes of data collection, dissemination, and storage; data sovereignty over shared data and its processing; and cultural assumptions regarding aspects such as personalization and surveillance.

Transparency, in this context, involves not only gaining insight into the technical features and processes of DCT apps but also understanding how these technologies operate, how they handle shared information, where such information is stored, and who has (or will have) access to it.

Data sovereignty is key in the context of a digital pandemic response, as individuals use DCT in a state of vulnerability. Safeguarding data sovereignty over one’s information can contribute to maintaining control over one’s information and thus consenting to the disclosure of sensitive data in the context of collective pandemic control.

In the “East-West Divide” section, we were able to demonstrate that experts perceive an East-West dichotomy. Western approaches are primarily viewed as individualistic and self-determined, whereas DCT approaches from East Asian countries such as China and Taiwan are considered collectivist and externally influenced.

### Limitations

However, at this point, it is necessary to acknowledge the limitations of this study, which should be addressed in future research endeavors. Furthermore, concerning our empirical analysis, we need to note limitations in the selection of materials and methods. Hence, our findings may have limited generalizability and transferability with respect to other DCT apps.

One methodological limitation was the use of a standardized questionnaire for all experts without considering their individual disciplinary perspectives. During our analyses, it became evident that an individualized questionnaire could have provided a deeper understanding and shed more light on the technical, societal, and legal dimensions. The main challenge here was the short project duration of 12 months, during which the team had to complete data collection, analysis, and publication. Additionally, this limited time frame coincided with the peak of the COVID-19 pandemic.

The qualitative data presented may not necessarily reflect representative findings. Nonetheless, we firmly believe that the aspects presented here, in conjunction with other relevant studies, are essential for current and potentially future pandemic response efforts. An empirical investigation to delve deeper into how the dimensions underlying DCT apps, such as individualism, freedom, surveillance, or privacy, embody sociocultural concepts would be a valuable undertaking. We have already published initial approaches to describe the algorithmization of culture in DCT apps elsewhere [12].

## Abbreviations

BMBF: Federal Ministry of Education and Research
CWA: Corona-Warn-App
DCT: digital contact tracing
ELISA: The Ethics of Live-tracking Applications in Connection with SARS-CoV-2
